# NF-κB in Alzheimer’s Disease: Friend or Foe? Opposite Functions in Neurons and Glial Cells

**DOI:** 10.3390/ijms252111353

**Published:** 2024-10-22

**Authors:** Barbara Kaltschmidt, Nele Johanne Czaniera, Wiebke Schulten, Christian Kaltschmidt

**Affiliations:** 1 Molecular Neurobiology, University of Bielefeld, 33615 Bielefeld, Germany; 2 Department of Cell Biology, University of Bielefeld, 33615 Bielefeld, Germany; nele.czaniera@uni-bielefeld.de (N.J.C.); wiebke.schulten@uni-bielefeld.de (W.S.); c.kaltschmidt@uni-bielefeld.de (C.K.); 3 Forschungsverbund BioMedizin Bielefeld, Ostwestfalen-Lippe (OWL) (FBMB E.V.), 33615 Bielefeld, Germany

**Keywords:** Alzheimer’s disease, transcription factor, NF-κB, TNF, DNA breaks, DNA damage repair, APOE4, neuroinflammation, glial cells

## Abstract

Alzheimer’s disease (AD) is a devasting neurodegenerative disease afflicting mainly glutamatergic neurons together with a massive neuroinflammation mediated by the transcription factor NF-κB. A 65%-plus increase in Alzheimer’s patients by 2050 might be a major threat to society. Hallmarks of AD are neurofibrillary tangles (NFTs) composed of hyperphosphorylated tau and amyloid beta (Aβ) plaques. Here, we review the potential involvement of transcription factor NF-κB by hereditary mutations of the tumor necrosis factor pathway in AD patients. One of the greatest genetic risk factors is *APOE4.* Recently, it was shown that the *APOE4* allele functions as a null allele in human astrocytes not repressing NF-κB anymore. Moreover, NF-κB seems to be involved in the repair of DNA double-strand breaks during healthy learning and memory, a function blunted in AD. NF-κB could be a friend to healthy neurons by repressing apoptosis and necroptosis. But a loss of neuronal NF-κB and activation of glial NF-κB in AD makes it a foe of neuronal survival. Hopeful therapies include TNFR2 receptor bodies relieving the activation of glial NF-κB by TNFα.

## 1. Introduction: Alzheimer’s Disease

Alzheimer’s disease (AD) was discovered by the German psychiatrist and neuropathologist Alois Alzheimer. In his first conference report (37th Conference of Southwest German Psychiatrists in Tübingen, 1906), he reported plaques and neurofibrillary tangles after histopathological examination of the brain of his long-term psychiatric patient Auguste Deter. Alzheimer later wrote: “Among the plaques of the cerebral cortex there were several of such enlarged size as I have never seen before, not even in the cornu ammonis of senile dementia” (translated from German, see [1]). Alzheimer described the senile plaques as amyloid plaques (“miliary foci”) together with neurofibrillary tangles (NFTs) [1]. Until today, these two biomarkers are still part of the neuropathological diagnosis.

## 2. Definition of Disease-Relevant Bits and Pieces

For consistence, we use the definitions of Serrano-Pozo and co-workers, 2011 [2].

**Figure 1:**
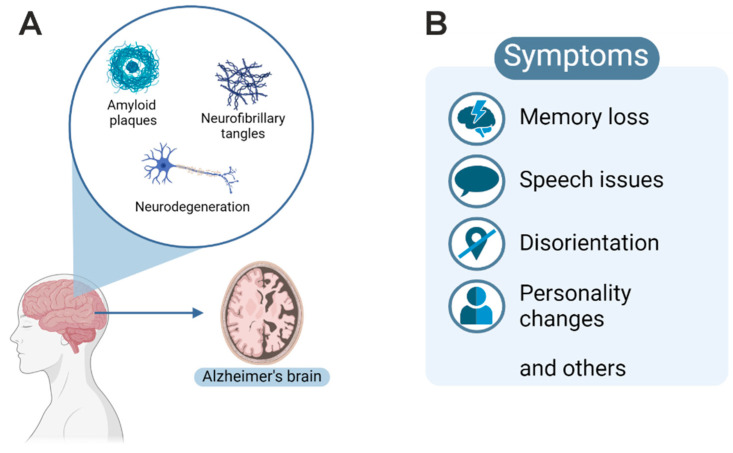
(**A**) Overview of histopathological features leading to cortical atrophy of the gyri with enlarged sulcal spaces (depicted as Alzheimer’s brain). (**B**) Various neurological symptoms, including memory loss, are associated with disease progression.

Aβ is the proteolytic product (40 or 42 amino acids) derived from the amyloid precursor protein (APP) by sequential cleavage of β- and γ-secretases. Tau is a bridging protein, stabilizing microtubules in axons. AD is characterized by translocation of tau to the somatodendritic segment, where hyperphosphorylation of tau could be driven by kinases such as protein kinase A [3]. Aggregation of hyperphosphorylated tau could drive the formation of NFTs and could interfere with axonal transport, leading to the increased formation of extracellular Aβ [3]. According to the Thal staging, amyloid plaques are extracellular Aβ aggregates that are often found in the late stages of Alzheimer’s disease [4]. They are usually classified as diffuse plaques without nuclei that are not correlated with microglial activation and synapse loss. In contrast, dense-core plaques have compact fibrillar Aβ deposits, positive for thioflavin S and Congo red staining. They are often surrounded by dystrophic neurites (neuritic plaques) together with activated microglia and astrocytes. Neuritic plaques are related to synaptic loss and cognitive impairment [2]. Changes leading to neurodegeneration are summarized in Figure 1. The two hallmarks of AD are depicted in Figure 1A as NFTs and Aβ plaques, leading to neurodegeneration (see degenerated neuron). Neurological symptoms include memory loss, speech issues, disorientation, personality changes, and others (see Figure 1B) [2].

New data suggest that bacterial lipopolysaccharide (LPS), presumably *E. coli*-derived endotoxin, which is a strong activator of NF-κB, is involved in AD [5]. More specifically, LPS seems to be contained within plaques [6]. Taken together, two activators of NF-κB: Aβ and LPS are present in plaques, which, in addition to neuronal dysfunction, could account for the massive neuroinflammation in later stages of AD. Currently, it is unknown if endotoxin stems from a Gram-negative bacterial brain infection or from the periphery.

## 3. Neuropathological Diagnosis

Today, modern clinical diagnosis could be supported by measurements of Aβ accumulation in vivo by PET scans [7]. Furthermore, a blood test with about 90% accuracy was developed, relying on measurements of Aβ and phosphorylated tau (p-tau217) [8]. Additional clinical tests are not the subject of this review.

But post-mortem neuropathological evaluation is still the gold standard for AD diagnosis [9] and is discussed below. Some of the macroscopic features of AD brains include cortical atrophy of the gyri with enlarged sulcal spaces. There is a loss of up to 50% of neurons, with volume loss mainly in the white matter [10], only slightly reflected in brain weight (normal brains: 1251 + 120 g; AD brains: 1061 + 163 g from DeTure and Dickson, 2019). Cortical thinning leads to enlargement of lateral ventricles during the cause of disease, which could be observed in MRI scans [11]. Surprisingly, more than 15% of the AD cases have additional diseases such as Lewy body type Parkinson’s disease. Microscopic features were initially described as plaques and tangles after silver impregnation by Alois Alzheimer. Heiko Braak and Eva Braak used a modified silver impregnation for a staging system of Alzheimer’s disease (Braak staging). Later on, a staging based on antibodies specific for phosphorylated tau protein was established by them [12], which detects neuronal lesions very early. In addition, there is a striking difference in the regional distribution of early stages, with phosphorylated tau starting in the locus ceruleus and Aβ pathology starting in the cerebral cortex. Thal from the Braak laboratory has further developed a staging using the anti-Aβ pathology [13]. Tau pathology leading to NFTs follows a stereotypic topographic distribution in different brain regions. It starts with lesions in the allocortex of the medial temporal lobe (entorhinal cortex and hippocampus) and disseminates in the associative isocortex, with relative spearing of sensory, motor, and visual areas. These topographic specializations, known as the Braak and Braak staging, are standard in neuropathological diagnosis [14]. In contrast to NFTs, Aβ plaques are first detected in great density in the isocortex. Later and to a lesser extent, other regions such as the allocortex (incl. the entorhinal cortex and the hippocampus) are involved [4]. In summary, the hippocampus CA1 and the entorhinal cortex are only involved at Thal phase 2. Taken together, it was hypothesized that the pathology associated with AD begins as tau pathology in selective, interconnected glutamatergic projection neurons of the association cortex. This phospho–tau accumulation might initiate Aβ formation by APP cleavage [3].

In five mouse AD models, it was shown that amyloid plaques are developed from so-called PANTHOS neurons [15]. These neurons contain Aβ in enlarged de-acidified autolysosomes, presumably due to low vATPase activity.

From a neuropathological point of view, NF-κB also plays a role in AD. Various studies have shown that the transcription factor is primarily active in neurons and glia cells, presumably astrocytes, but not in microglia [16,17,18,19,20,21]. In Table 1, we summarize the neuropathological findings in AD associated with NF-κB.

## 4. Biomarkers and Genetics

### 4.1. Biomarkers

Hyperphosphorylated tau is a biomarker that is detectable very early, even in disease-free stages. Late stages of tau pathology are seen in more than 20% of patients older than 70 years [3]. Another historically early-discovered biomarker is Aβ, a main component of amyloid plaques. Surprisingly, plaques were also detected in brains of Down syndrome (trisomy 21) patients. Konrad Beyreuther purified and sequenced Aβ from Down syndrome patients. Later on, he teamed up with Colin Masters and purified plaques from Alzheimer’s patients’ brains. He solubilized the plaque components with formic acid and obtained similar protein sequences, as he already described for Down syndrome [22]. Together with Benno Müller-Hill, Beyreuther succeeded in cloning the amyloid precursor molecule (APP) [23]. This shows that Aβ is a 4 kDa fragment of APP produced by many neurons of the brain, by vascular cells and blood cells, but to a lesser extent by astrocytes. Aβ pathology with fibrillary Aβ was detected in more than 50% of patients over 70 years [3].

### 4.2. Genetics

**Figure 2:**
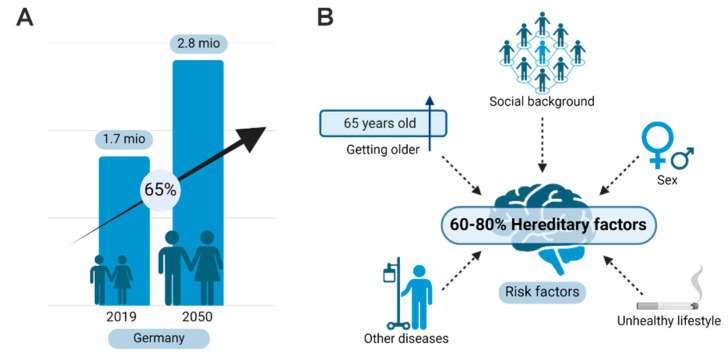
Alzheimer’s disease is a major threat for society. (**A**) Data from Germany are plotted as number of cases and show a tremendous increase in AD for the next 30 years. (**B**) Major non-genetic risk factors and hereditary risk factors contribute to the development of AD.

A 65% increase in cases from 2019 to 2050 is expected only in Germany [24]. Genetics are very strong, as hereditary factors were considered at 80% among age, social background, female sex, or unhealthy lifestyle (Figure 2A,B) [25].

Here, we will shortly review genetics involved in Alzheimer’s disease. Missense mutations within the gene of *presenilin1* (*PSEN1*) on chromosome 14 are most frequent in early-onset AD. A genetic analysis showed that mutations of *PSEN1* and *APP* accounted for 71% of families with early-onset AD (younger than 61 years), whereas mutations in *PSEN2* were lower in frequency [26]. Furthermore, trisomy 21 (Down syndrome) with *APP* on chromosome 21 shows an AD-like pathology with amyloid plaques in the early twenties of life and neurofibrillary tangles starting at an age over 20. In addition to the gene dosage and the effect of an additional *APP* copy on chromosome 21, other genes on the same chromosome could also play a role in the development of Alzheimer’s disease in patients with Down syndrome [27].

A recent genome-wide association study (GWAS) [25] analyzed more than 100,000 clinically diagnosed AD patients. More than 600,000 controls revealed a list of genes with a highly significant correlation to AD. Among the genes with the highest significances are *APOE* (highest *p* value *p* < 10^−118^); *BIN1* (*p* value 6 × 10^−118^); *CR1* (*p* value 7 × 10^−46^); *TREM2* (several SNPs up to a *p* value 3 × 10^−37^); *CLU* (*p* value 2 × 10^−44^); *PICALM* (*p* value 3 × 10^−48^); and *MS4A* (*p* value 4 × 10^−42^). For comparison, the *p*-value of *APP* is 1 × 10^−12^ in this study [25]. Next, this genetic study revealed several loss-of-function mutations in genes upstream in the NF-κB pathway, such as *ADAM17*, *SHARPIN*, *TNIP*, *HOIL*, and *OTULIN.* All these gene products might be involved in the neuronal TNF signaling, as reviewed in [28]. Furthermore, the sortilin-related receptor 1 (*SORL1*, *SORLA*) gene was shown to be strongly associated with Alzheimer’s disease [29]. In addition, the AD risk gene *ABCA7* can be found in up to 7% of AD patients [30]. Interestingly, *ABCA7* haplodeficiency drives excessive microglial Aβ accumulation, with statistically significant lower NF-κB activation after LPS stimulation [31].

A recently performed observational study of chronic autoimmune inflammatory diseases and risk of AD using more than 2 million patients’ data from the UK identified an increased risk for AD correlated with elevated pro-inflammatory cytokines in circulation, such as IL6, IL1α, and β, but not TNFα and β [32]. *TNFα* G308A polymorphism was been reported as a suscebility gene for AD, but recent metanalysis restricted this observation to the Chinese population [33]. But a recent study showed increased levels of TNFα in the serum of AD patients [34]. In addition, a short-term anti-TNFα treatment could improve cognition in AD patients [35]. Interestingly, increased levels of TNFR1 in serum might predict a conversion of mild cognitive impairment to AD [36]. Furthermore, transmembrane protein TREM2, expressed in microglia and driving an anti-inflammatory gene expression program, was identified as a risk factor in AD [37].

In the following, we will summarize expression data from the human protein atlas for significant AD-related genes (see Table 2).

**Figure 3:**
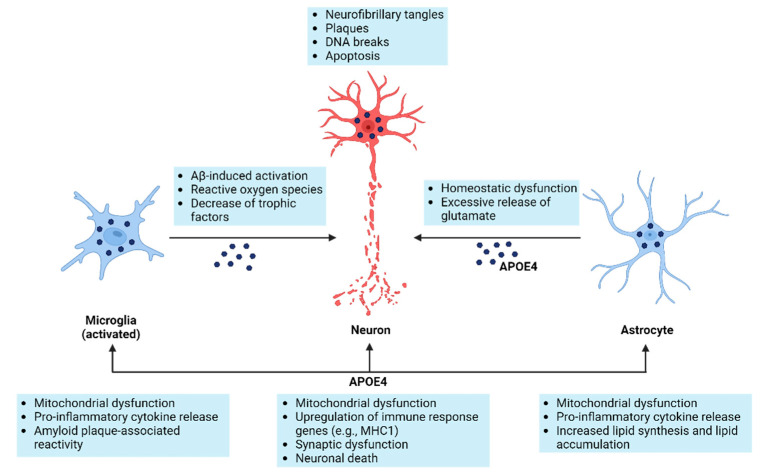
Overview of neuroinflammation in AD and the impact of APOE4 on neurons and glial cells.

*APOE* is very well known to bear a risky allele for AD (*APOE4*). APOE is a 299-amino-acid glycoprotein, which could function as a lipid transporter for cholesterol and phospholipids. It is highly expressed in neurons and astrocytes of the cerebral cortex and hippocampus and to a lesser extent in microglia (see Figure 3). A recent study describing human iPS cells differentiated into astrocytes investigated *APOE* genotypes [38]. In contrast to *APOE3* the *APOE4*, an Alzheimer’s-risk gene drives astrocytes into exacerbated inflammation with a high expression of IL-6 and IL-8. They presume a loss of the potential NF-κB inhibitor TAGNL3 as a molecular mechanism. CR1 is a complement receptor with highly selective protein expression in immune cells such as macrophages and B-cells. BIN1 is an adaptor protein, which might be involved in synaptic vesicle trafficking in neurons but also in the regulation of MYC activity. Patient specific, it is highly expressed in glia and neurons of the hippocampus. TREM2 can act as an Aβ 42 receptor for uptake and degradation within microglia. Furthermore, it is a member of the macrophage and microglia immune response cluster. CLU is an extracellular chaperone preventing aggregation of non-native proteins, such as the formation of amyloid fibrils. Expression seems to be mainly in astrocytes. PICALM is a phosphatidylinositol-binding clathrin assembly protein with a potential role in endocytosis. It is mainly expressed in oligodendrocytes within the brain. MS4A is a membrane-spanning 4-domains protein with no orthologous mouse protein. It is mainly expressed in microglia. APP is exclusively expressed in neurons localized in cell membranes and the cytoplasm. It is a target gene of NF-κB [39]. Tau (MAPT) is expressed in various isoforms in neurons and bone cancer; its function is in microtubule stabilization and polarization. It is mainly localized within the neuropil in a normal brain and found aggregated and phosphorylated in AD, even at early stages.

Therefore, we think a lot more should be done for the functional understanding of AD. Indeed, novel studies use novel tools (iPS or direct reprogramming) for functional studies with human cells. We will review these in the following section.

## 5. Cells Involved in Alzheimer’s Pathology

Finally, AD is a neurodegenerative disease, which might not mean that there is no crucial involvement of glial cells. Indeed, there seems to be a massive neuroinflammation and microglial activation [40] within Alzheimer’s patients’ brains. Innate immune cells within the brain, such as microglia and astrocytes, initiate a neuroinflammatory response leading to neurodegeneration with crucial involvement of the *APOE4* allele [41] (see Figure 3). Typically, pro-inflammatory cytokines such as TNFα, IL-1β, and IL-8 are secreted by activated microglia. Furthermore, an increase in reactive oxygen species and loss of neurotrophins (trophic support) might further exaggerate neurodegeneration. There are two signaling pathways within neurons competing after TNF stimulation: a neuroprotective signaling regulated by NF-κB and an apoptotic one driven by caspase 8. When NF-κB is inhibited, pro-apoptotic signaling wins, and apoptosis might be the consequence [42]. A recent study describing human iPS cells differentiated into astrocytes investigated *APOE* genotypes [38]. In contrast to *APOE3*, *APOE4*, an Alzheimer’s risk gene, drives astrocytes into exacerbated inflammation with a high expression of IL-6 and IL-8. They presume a loss of the potential NF-κB inhibitor TAGNL3 as a molecular mechanism.

## 6. NF-κB

**Figure 4:**
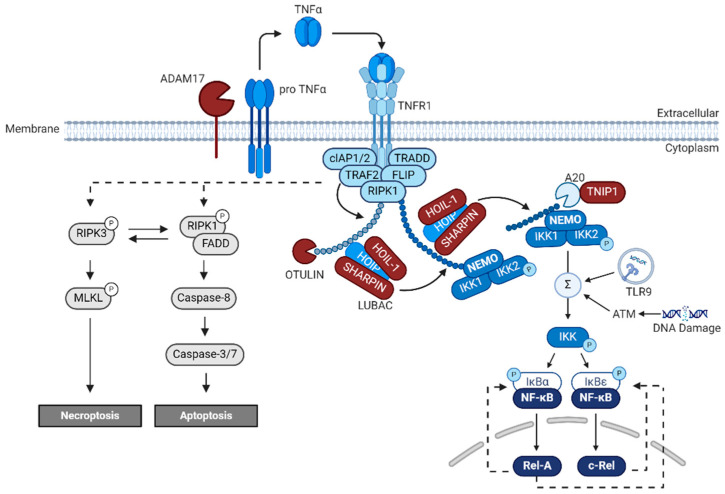
Overview of TNFR1-mediated activation of NF-κB dependent on linear ubiquitin. Membrane-bound TNF could be released by proteolytic cleavage mediated by ADAM17, resulting in intracellular signaling dependent on linear ubiquitin (small blue pearls). Activation of trimeric IKK complex could trigger nuclear localization of REL or RELA, which are controlled by feedback loops with IκBα or IκBε (modified from Sasaki et al., 2023 [53]). Alternatively, when ubiquitination of RIPK 1 is inhibited, interaction with FADD occurs, leading to activation of CASPASE-8-mediated cell death. In contrast, if CASPASE-8 is inhibited, RIPK3 interacts with MLKL, leading to necroptosis.

The mainly positive acting transcription factor NF-κB is thought to be a key regulator of inflammation and cancer progression and vitally drives a broad range of cellular processes within the mammalian nervous system [28]. Many extracellular factors activating NF-κB in the nervous system are active, such as Aβ [43], the pro-inflammatory cytokine tumor necrosis factor (TNFα) [19,44], the neurotransmitter glutamate [45,46] and its agonists like kainate [46], and N-methyl-D-aspartate (NMDA) [47]. Here, we will review a potential role of NF-κB in neuroprotection and neuroinflammation during AD. The NF-κB family of transcription factors is composed of five DNA-binding members with the following HUGO gene symbols in humans: *REL (c-REL)*, *RELA (p65)*, and *RELB (RELB)*, as well as *NFKB1 (p50)* and *NFKB2 (p52)*, with the latest two lacking transactivation domains [48]. Classically, NF-κB-signaling is divided into the canonical, non-canonical, and atypical pathways. Canonical and non-canonical NF-κB-signaling share a common regulatory element, the inhibitor of κB (IκBα) and the IκB kinase (IKK). In non-canonical NF-κB-signaling, ligand binding to receptors like CD40 activates IKK1 via NF-κB-inducible kinase (NIK). Phosphorylation of IKK1 results in processing of p100 to p52 and subsequent nuclear translocation of p52/RELB [49]. NF-κB dependent gene expression comes in several distinct time-dependent programs, such as constitutive activity, inducible activity, and subunit activity, and are activated and repressed in a large time scale [50]. Canonical NF-κB-signaling could be started by trimerization of TNFR1 by its trimeric ligand TNFα [51] (Figure 4). Trimerization induces formation of an intracellular signaling complex composed of adaptor proteins such as the TNFR1-associated death domain (TRADD), receptor- interacting Ser/Thr-protein kinase 1 (RIPK1), and E3 ligases such as TNF receptor-associated factor 2 (TRAF2) and cellular inhibitor of apoptosis proteins 1 and 2 (cIAP1 and cIAP2) [52]. E3 ligases as above use the N-terminal methionine (M1) of the proximal ubiquitin for linear polyubiquitin chain elongation [53]. Polyubiquitin chains on RIPK1 could recruit the LUBAC complex that catalyzes linear ubiquitination. The LUBAC complex is composed of at least three different components: heme-oxidized IRP2 ubiquitin ligase 1 L (HOIL-1L), the HOIL-1L-interacting protein (HOIP), and the SHANK-associated RH domain-interacting protein (SHARPIN). Recently, two patients with homozygous SHARPIN deficiency were identified [54] that depicted clinical inflammatory features in contrast to individuals with HOIL-1 and HOIP deficiencies. Surprisingly, the human disease did not recapitulate the severe dermatological phenotype of SHARPIN-deficient mice. Another negative regulator of the LUBAC complex is OTULIN [53]. By LUBAC deficiency or cIAP inhibition, TNF stimulates cell death-inducible complex II, which induces apoptosis (see Figure 4). Furthermore, A20 down-regulates LUBAC-induced NF-κB activation by specifically binding to the M1-ubiquitin chain through the zinc finger 7 (ZF7) domain [55]. This A20-NF-κB repressing activity is counteracted by TNIP1 (ABIN1). *TNIP1*^−/−^ knockout mice died 20 weeks after birth with massive systemic inflammation [56].

Activation of NF-κB in neurons by TNFR1 containing a death domain could prevent apoptosis by TNF only, when NF-κB dependent transcription of *FLIP-L* is initiated [42]. FLIP-L could inhibit the pro-apoptotic cytoplasmic complex II containing caspase 8/10. But when caspases are not active, RIPK3 and MLKL are recruited and necroptosis is initiated (see Figure 4) [57]. Surprisingly, neuronal necroptosis is a major form of regulated cell death in AD [58]. Thus, translocation of phosphorylated MLKL oligomers to the cytoplasmic membrane forms pores releasing intracellular contents, ultimately leading to necroptotic cell death. Surprisingly, there seems to be a switch from the neuronal expression of TNFR2 without death domain to the expression of TNFR1 with death domain as reviewed [58]. Markers of necroptotic cell death, such as phosphorylated MLKL, were particularly high in degenerating neurons within AD brains [59].

Finally, phosphorylation of the NEMO/IKK1/IKK2-complex and release from TNFR1 is mediated (Figure 4). Phosphorylated IKKs in turn phosphorylate IκBα and IκBε, which kept p65/p50 in an inactive cytoplasmic state [60]. IKK-dependent phosphorylation of IκBα results in its proteasomal degradation and enables nuclear translocation of dimeric complexes containing REL or RELA and expression of target genes (Figure 4), controlled by feedback loops with IκBα or IκBε.

## 7. Small Molecule Drugs and Biologicals

There are many small drugs directly inhibiting NF-κB activation by inhibition of the IKK complex, but none made it to clinical application [61]. Clinical application of three NF-κB-inhibiting drugs as frequently used for the therapy of multiple myeloma are glucocorticoids, proteasome inhibitors, and thalidomide derivates. Here, we discuss the effect of these drugs on AD patients.

### 7.1. Small Molecule Drugs

Long-term systemic glucocorticoid usage was associated with significant atrophy of the hippocampus and amygdala, as a recent meta-analysis reported [62]. But a register study of German health insurance data revealed a significant reduction in dementia for users of glucocorticoids. Surprisingly, the lowest hazard ratio (HR) was reported for inhaled glucocorticoids (HR = 0.65, CI = 0.57–0.75) and users of nasal application (HR = 0.76, CI = 0.66–0.87). Even oral application could reduce the incidence of dementia (HR = 0.83, CI = 0.78–0.88) [63]. The ubiquitin proteasome system seems to be severely hampered in AD patients by the presence of Ubi + 1 mutant ubiquitin forms, which interfere with the normal proteasome degradation [64]. Furthermore, proteasome activity showed a large interpersonal variation [65], where low activity could lead to AD. Thalidomide treatment in AD patients showed bad tolerability; the therapeutic dose could not be reached and the study had to be abrogated [66]. Furthermore, a Cochrane metanalysis reported no evidence to support the use of low-dose aspirin or other NSAIDs of any class (such as celecoxib, rofecoxib, or naproxen) for the prevention of dementia, but there was evidence of harm [67]. In this line, a recent review concentrated on NF-κB inhibitors for therapeutic use in AD. Only in vitro or animal studies were reported, but no clinical use of small molecules inhibiting NF-κB were reported [68]. For glial inhibition of NF-κB, the drug minocycline might be useful [69]. In a recent clinical trial named MADE, it was shown that, in patients with mild AD, minocycline had no effect on cognitive decline [70].

### 7.2. Biologicals

TNF could be inhibited by various biologicals such as etanercept, infliximab, and adalimumab; clinical trials were summarized by a recent meta-analysis [71]. Surprisingly, treatment with anti-TNF biologicals used for other diseases, such as rheumatoid arthritis, had a large beneficial effect. Thus, three large epidemiology studies reported that anti-TNF antibody-treated patients had a 60 to 70% lower odds ratio compared to untreated. It is discussed that the effect is on TNF outside the nervous system. Surprisingly, the best effects are reported for etanercept, a biological where the ligand binding domain of TNFR2 was fused to the Fc of antibodies. In this line, etanercept at 25–50 mg, once weekly, was administered by a new application route: peri spinal administration with placement of the head below a horizontal position to increase transport into the spinal fluid. In this study, a hopeful result was presented: a significant improvement, measured by all of the primary efficacy variables, through 6 months [35].

## 8. Mouse Models for NF-κB Function in Learning and Memory and AD

In the beginning of the 21st century, several new transgenic mouse models with specific repression or activation of NF-κB in the nervous system were generated, allowing studies of behavior and learning, as reviewed in Kaltschmidt et al., 2022 [28]. Mollie Meffert and coworkers discovered that *p65*-deficient mice survive when crossed to *TNFR1*-deficient mice. These mice had a learning defect of spatial learning in a radial arm maze and had no synaptic NF-κB [47]. We discovered that expression of transdominant negative IκBα in glutamatergic neurons affected spatial memory formation and repressed expression of the novel NF-κB target gene *protein kinase A (catalytic subunit α)* [72]. Warner Greene and coworkers discovered that neuronal expression of super-repressor IκB in GABA-ergic interneurons led to enhanced spatial learning and memory [73]. Later on, it was shown that NF-κB-repression by transdominant negative IKK2 led to impaired learning and memory. The authors further identified *insulin-like growth factor 2 (IGF2)* as a novel IKK/NF-κB target gene. In this line, IGF2 was able to restore synapse density and promoted spine maturation in IKK/NF-κB signaling-deficient neurons within 24 h [74]. In contrast, expression of a constitutively active allele of *IKK2* in forebrain neurons led to degeneration of microglia and astrocytes as well as spatial learning defects [75]. Therefore, evidence indicates NF-κB involvement in learning and memory in healthy neurons and additionally in brain regeneration in vivo.

Recently, NF-κB in neurons was analyzed in two models of AD, leading to conflicting results. Whereas inhibition of NF-κB by *IKKβ* deficiency was protective against neuronal death in an Aβ model, it was not in a model with phosphorylated tau [76].

An inducible *CK-p25* mouse model for AD [77] leads to aberrant activation of cell cycle kinase Cdk5, generating a phenocopy of Alzheimer’s disease with hyperphosphorylated tau and neuronal loss in the cortex and hippocampus followed by an Alzheimer’s-disease-like forebrain atrophy, including intracellular Aβ accumulation. Using *CK-p25* mice, it was detected that neurons with double-strand breaks activate a transcriptional program of innate immunity reminiscent to senescent cells [78]. Furthermore, knockdown of *RELA* activity in neurons rescued synaptic loss and interfered with microglial activation and proliferation.

As a note of caution: it has been shown that the inflammatory response is quite different in mice and men [79]. Better animal models for AD might be aged rhesus monkeys [3], but these must be aged for 18 to 30 years, and there is no system for easy genetic manipulations in addition to ethical considerations. Therefore, from our point of view, the best currently available model system for AD might be human iPS cells with isogenic controls or brain organoids.

## 9. DNA Double-Strand Breaks in AD

In living cells, DNA is subject to a constant process of oxidative damage by oxygen free radicals (reactive oxygen species—ROS) that are produced inside the cell as a result of metabolic processes [80,81]. It is estimated that in a single cell cycle, at least 5000 single-stranded DNA breaks can occur as a result of ROS production. Approximately 1% of these DNA breaks is converted into double-strand breaks (DSBs), mainly during DNA replication, while the remaining 99% is repaired. Thus, during the cell cycle in a single nucleus, about 50 so-called “endogenous” DSBs are formed. Accumulation of unrepaired DNA damage induced by ROS leads to cell aging and may be responsible for the induction of neoplastic transformation [80,82]. Histone H2AX is a substrate of several phosphoinositide 3-kinase-related protein kinases (PIKKs), such as ATM (ataxia teleangiectasia mutated), ATR (ATM and Rad3-related), or DNA-dependent protein kinase (DNA-PK). ATM kinase is considered as a major physiological mediator of H2AX phosphorylation in response to DSB formation [83,84]. DNA damage is a marker for aging and neurodegeneration [85,86]. Furthermore, DNA damage repair pathways are actively transcribed in the aging brain, and age-associated neurodegenerative diseases depict DNA lesions and reduced DNA repair efficiency [87,88,89]. DNA double-strand breaks are the most deleterious processes, since they can drive many phenotypes of aging, including senescence, mutations, and cell death. Especially postmitotic neurons are sensitive to these threats due to their long life span, high metabolic activity, and limited DSB repair capacity. Furthermore, the accumulation of DSBs is an early feature of AD, suggesting that they may act as an initiating lesion of toxicity [90].

Recently, two distinct populations of neurons were described when analyzing gene expression induced days after fear conditioning [91]. These include engram neurons with immediate early gene transcription, such as *CREB*, *cFOS*, *Egr1*, etc., and inflamed neurons making *IL-6*.

Signaling within inflamed neurons is mediated by TLR9 detecting extra-nuclear fragments of DNA in endolysosomes (Figure 4). RELA is needed for this type of DNA damage repair to be localized to the centrosome, where the protein 53BP1 could mediate DNA repair. Mice with knockout of either *TLR9* or *RELA* were unable to retrieve fear-associated memories, suggesting that both engram neurons and inflammatory neurons are essential for memories to persist.

In humans with AD, the number of DNA breaks is increased [92]. Furthermore, DNA damage repair involving ATM, which might activate NF-κB, is blunted [93]. In induced neurons (iN) produced in vitro by regulated expression of transcription factor *NEUROG2* and *ASCL1* from AD patients, strikingly different results were described [94]. Gene sets linked to NF-κB were significantly upregulated (*p* = 0.0329) when fibroblasts were used for iN but not when iPS cells were used (*p* = 0.7024) [94]. Finally, activated NF-κB is reduced in neurons around major plaque stages [19].

## 10. Conclusions

**Figure 5:**
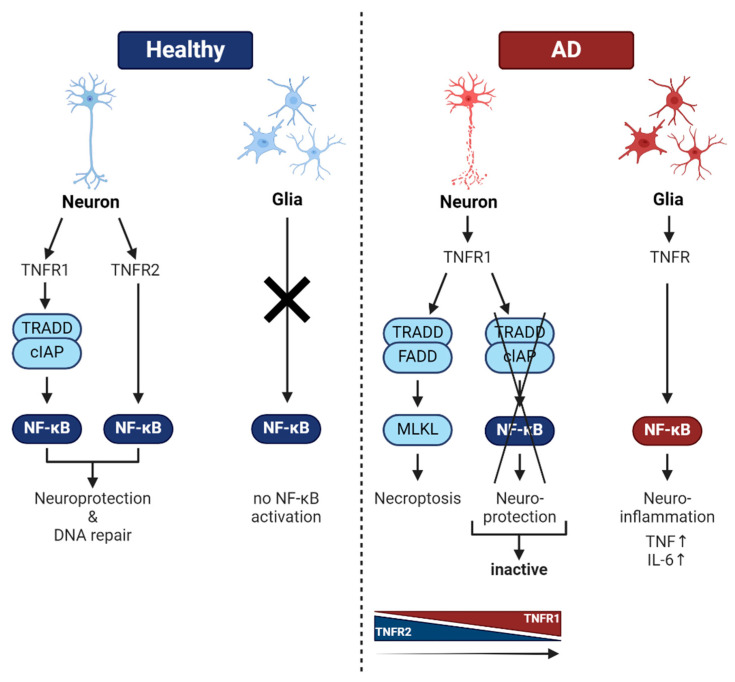
Summary of NF-κB function in healthy brain and AD. On the left side (in blue), the function of NF-κB in healthy neurons is depicted. Signaling through TNFR1 and 2 could lead to the expression of a neuroprotective gene program, inhibiting apoptosis as well as DNA repair. Glial cells do not contain activated NF-κB under healthy conditions. On the right side, NF-κB activity in AD is depicted (in red). Two main cell types are afflicted: neurons and glia. In contrast to healthy tissue, mainly TNFR1 is involved, without neuronal NF-κB activation, leading to neuronal death by necroptosis. In glial cells, a chronic inflammation is regulated by TNFR-mediated NF-κB activation, leading to high expression of proinflammatory cytokines TNF and IL-6. These could further exacerbate neuronal death.

NF-κB in healthy neurons is involved in neurotransmission, learning, and memory. In this context, repair of DNA double-strand breaks dependent on NF-κB might be crucial for learning and neuronal integrity. In contrast, during AD glial inflammation, regulation by NF-κB might exacerbate intrinsic neurodegeneration, triggered by phosphorylated tau and Aβ (see Figure 5, right side). On the other hand, a neuronal TNF-mediated protection program relying on activated NF-κB might be essential for neuronal survival under stress (ROS, DNA-damage, pro-inflammatory cytokines, glutamatergic neurotransmission, etc.; see Figure 5, left side). This TNF signaling pathway might be dysfunctional in AD patients by hereditary mutations. Furthermore, there is a switch from TNFR2 expression in healthy brains to mainly TNFR1 expression in brains of AD patients, leading to necroptosis [58]. Taken together, NF-κB seems to be beneficial to neurons, whereas glial activation of NF-κB is a foe. Still, a lot of open questions remain, such as the involved NF-κB subunits/genetic variants, the interplay of glia and neurons, the target genes of NF-κB (gene signature) for healthy and diseased AD neurons, and, finally, the therapy. In this line, a beacon of hope for AD therapy might be biologicals such as TNFR2 receptor bodies.

## Figures and Tables

**Table 1:** Summary of neuropathological findings in AD.

Finding	Antibody	Case Number	References
strong IR in neurons in AD, much lower in controls whole hippocampus formation extremely high and mainly nuclear in CA3 prominent staining in neuronal processes as well as neurofibrillary tangles and dystrophic neurites staining of astrocytes no microglial staining	anti-RELA	7 non-AD 7 AD	[16]
65% of plaques surrounded by cells with activated nuclear RELA pyramidal neurons and glia surrounding plaques, presumable astrocytes, not in activated microglia, strongest staining in primitive plaques	anti-RELA-NLS mAb	4 AD	[17]
highly enriched nuclear RELA in cholinergic neurons of AD (nucleus basalis of Meynert)	RELA		[18]
RELA in cells around plaques, graded loss of RELA IR during plaque maturation from primitive to classical plaques, lower IR in AD compared to controls in early plaque stages	anti-RELA-NLS mAb		[19]
enhanced expression of IκBα in the cytoplasm of degenerating neurons with tangle formation REL nuclear in CA1 neurons with tangles (AT8 double pos.) southwestern blotting revealed strong binding of kB motif dsDNA to neuronal elements in AD but not in controls RELA mainly nuclear but p50 mainly cytoplasmic	anti-IκBα, anti-p50 anti-RELA anti-REL AT8		[20]
nuclear P-RELA highly enriched (>10-fold) in GFAP + astrocytes in AD compared to non-AD, p50 not nuclear			[21]

**Table 2:** Cell-type-specific expression and function of genes mutated in AD. Data were extracted from the protein atlas (www.proteinatlas.org (accessed on 7 October 2024)).

Gene	Function	Cell Type
*APOE*	lipid transporter	neurons and astrocytes
*CR1*	complement receptor	macrophages and B-cells
*BIN1*	adaptor protein	neurons
*TREM2*	Aβ42 receptor	microglia
*CLU*	chaperone	astrocytes
*PICALM*	Phosphatidylinositol-binding clathrin assembly protein	oligodendrocytes
*MS4A*	Membrane-spanning 4-domains protein with no orthologous mouse protein	microglia
*APP*	Aβ precursor protein	neurons
*Tau (MAPT)*	microtubule-associated protein	neurons
